# On the molecular mechanism of GC content variation among eubacterial genomes

**DOI:** 10.1186/1745-6150-7-2

**Published:** 2012-01-10

**Authors:** Hao Wu, Zhang Zhang, Songnian Hu, Jun Yu

**Affiliations:** 1 James D. Watson Institute of Genome Sciences, Zhejiang University, Hangzhou 310007, China; 2 CAS Key Laboratory of Genome Sciences and Information, Beijing Institute of Genomics, Chinese Academy of Sciences, Beijing 100029, China

## Abstract

**Background:**

As a key parameter of genome sequence variation, the GC content of bacterial genomes has been investigated for over half a century, and many hypotheses have been put forward to explain this GC content variation and its relationship to other fundamental processes. Previously, we classified eubacteria into dnaE-based groups (the dimeric combination of DNA polymerase III alpha subunits), according to a hypothesis where GC content variation is essentially governed by genome replication and DNA repair mechanisms. Further investigation led to the discovery that two major mutator genes, *polC *and *dnaE2*, may be responsible for genomic GC content variation. Consequently, an in-depth analysis was conducted to evaluate various potential intrinsic and extrinsic factors in association with GC content variation among eubacterial genomes.

**Results:**

Mutator genes, especially those with dominant effects on the mutation spectra, are biased towards either GC or AT richness, and they alter genomic GC content in the two opposite directions. Increased bacterial genome size (or gene number) appears to rely on increased genomic GC content; however, it is unclear whether the changes are directly related to certain environmental pressures. Certain environmental and bacteriological features are related to GC content variation, but their trends are more obvious when analyzed under the dnaE-based grouping scheme. Most terrestrial, plant-associated, and nitrogen-fixing bacteria are members of the dnaE1|dnaE2 group, whereas most pathogenic or symbiotic bacteria in insects, and those dwelling in aquatic environments, are largely members of the dnaE1|polV group.

**Conclusion:**

Our studies provide several lines of evidence indicating that DNA polymerase III α subunit and its isoforms participating in either replication (such as *polC*) or SOS mutagenesis/translesion synthesis (such as d*naE2*), play dominant roles in determining GC variability. Other environmental or bacteriological factors, such as genome size, temperature, oxygen requirement, and habitat, either play subsidiary roles or rely indirectly on different mutator genes to fine-tune the GC content. These results provide a comprehensive insight into mechanisms of GC content variation and the robustness of eubacterial genomes in adapting their ever-changing environments over billions of years.

**Reviewers:**

This paper was reviewed by Nicolas Galtier, Adam Eyre-Walker, and Eugene Koonin.

## Background

As one of the key parameters of genome sequences, the genomic GC content, confined to between 25% and 75%, has been investigated for over half a century [1-3]. There are several essential questions to be addressed concerning GC content and its variability. First, how does it vary: randomly, gene-centrically, species-specifically, regulated, or selected? Second, at what level does GC content vary: replication, transcription-coupled, or functionally selected (proteins)? Third, what are the outcomes or biological significances of GC content variability: thermostability, protein-coding requirement, or biased mutations? Fourth, could GC content be changed *in vitro *globally or locally in terms of genes and genomes? It is obvious that we have very limited knowledge of how a genome ends up with a particular GC content.

Codon usage bias, especially GC content at the third codon position, correlates with the trend of GC content variations [4], and accumulating evidence indicates that it may be selected by gene expression [5-7]. Therefore, it has been proposed that codon usage bias may be driven by GC content changes, but not vice versa [8,9]. Mutations should generally conform to two patterns--global or transcript-centric--each derived from different mechanisms. The former is attributable to DNA replication and global repair and the latter is mainly the result of transcription-coupled repair [10-12]. Concerning the fundamental role of the environment or habitat in species evolution [13-15], another way to study GC content variation is to differentiate intrinsic from extrinsic (mostly environmental) factors, and to measure their impacts on GC content variability and evolvability, both qualitatively and quantitatively. Different hypotheses have been proposed by numerous authors to explain why GC content varies and how it is related to different intrinsic and extrinsic factors [16-28].

To better understand the relationship between GC content variation and mutational mechanisms, we attempted to correlate global GC content changes with DNA replication and repair, focusing on prokaryotes [28-30]. We discovered an excellent correlation between GC content variations and the dimeric combinations of DNA polymerase III alpha subunits, which showed that eubacteria can be grouped into different GC variable groups: the full-spectrum or dnaE1 group, the high-GC or dnaE2-dnaE1 group, and the low GC or polC-dnaE3 group [28]. We have extended our analyses into several mutator genes [31,32] to further elucidate the potential mechanisms.

In this study, we analyzed GC content variability based on a comprehensive evaluation of its relationship to various intrinsic and extrinsic factors, as well as an in-depth investigation of the translesion synthesis (TLS) pathway and its relevant mutator genes. The results indicated that replication and SOS mutagenesis are the major processes affecting GC content, and other environmental or bacteriological factors, such as genome size, temperature, oxygen requirement, and habitat, either play subsidiary roles or indirectly rely on different mutator genes to alter the GC content. Our results provide a comprehensive insight into the robustness of eubacterial genomes in adapting to their ever-changing environments through a basic composition parameter change--the GC content.

## Results

### GC content variations in the three dnaE-based eubacterial groups

**Figure 1:**
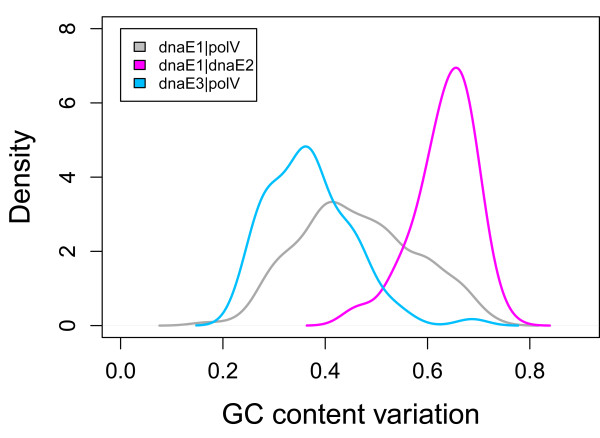
**GC content distribution in three dnaE-based groups**. The grouping of dnaE1|polV, dnaE1|dnaE2, and dnaE3|polV are based on a collection of 364 non-redundant bacteria.

For the convenience of discussion, we summarized 10 hypotheses as potential reasons for generating GC content variation (Table 1) [16-28]. While we admit that our collection is not comprehensive, it provides useful examples and a basis for discussion. The study of GC content variation focused on a dataset containing 364 non-redundant eubacterial genomes, rather than all of the bacterial genomes available in the public databases (see Materials and Methods). We use a dnaE-based grouping scheme to guide our analysis, which is based on the presence and absence of different PolIII (Polymerase III) alpha subunit isoforms, as defined previously [28,29]. To include the two key mutator genes, *dnaE2 *and *polV*, we renamed the groups as dnaE1-dnaE1|polV, dnaE1-dnaE1|dnaE2, and polC-dnaE3|polV, which for convenience we abbreviated as dnaE1|polV, dnaE1|dnaE2, and dnaE3|polV, respectively. The 364 eubacterial genomes were thus classified into the three groups: 173 in dnaE1|polV, 115 in dnaE1|dnaE2, and 76 in dnaE3|polV. The two mutator genes, *dnaE2 *and *polC*, are likely to play different roles in GC content variation. *dnaE2*, a well-known mutator gene, strongly correlates with high GC content (Figure 1). *polV *is also assumed to be heavily involved in GC content variation because its presence in the two groups lacking *dnaE2 *is closely related to either GC content variability (in the dnaE1|polV group) or GC content constraint (in the dnaE3|polV group). The presence of *polC *correlates with low GC content.

**Table 1:** Hypotheses proposed to explain GC content variations in eubacteria

Hypotheses	Time	Content	Reference
UV resistance	1970	Since ultraviolet radiation induces the formation of thyminedimers, higher GC content gives a selective advantage to organisms living in niches that are susceptible to direct and intense sunlight.	16,17
Thermal adaptation	1984	Thermophilic organisms demonstrate a tendency to high GC content because thermostable and thermolabile amino acids are encoded by GC-rich and GC-poor codons respectively.	18, 19
AT to GC mutation	1988	Practically all organisms are subjected to directional mutation pressure and this offers plausible explanations for the intensive GC content heterogeneity among different chromosomal regions of vertebrate genomes.	20
Metabolic resource	1995	Differences in directional nucleotide substitution among lineages of mammals can be explained by changes in metabolic physiology. This relationship is thought to be mediated by the effect of oxygen radicals.	21
Coding sequence length	1996	The longest coding sequences (exons) of vertebrates and genes of prokaryotes are more GC-rich than the shortest ones.	22, 23
Nitrogen-fixation	1998	There is a significantly higher GC content in the nitrogen-fixing members of the genus than in those unable to fix nitrogen.	24
Oxygen requirement	2002	Aerobic prokaryotes display a significant increment in genome GC% in relation to anaerobic ones.	25
Environment pressure	2005	The GC content of complex microbial communities seems to be globally and actively influenced by the environment, such as bacteria in surface water samples having a GC-content median of around 34%, while for soil samples, it is around 61%.	26
Genome size	2006	The relationship between genome size and GC level is valid for aerobic, facultative, and microaerophilic species.	27
DNA polymerase III	2007	According to the dimeric combination of alpha subunits, GC contents of eubacterial genomes are partitioned into three groups with distinct GC content variation spectra: dnaE1 (full-spectrum), dnaE2/dnaE1 (high-GC), and polC/dnaE3 (low-GC).	28

### Bacteriological features among the dnaE-based eubacterial groups

We explored the correlation between our grouping scheme (which is largely GC content-related and mechanism-based) and a variety of bacteriological features, including oxygen requirement, temperature, habitat, and several metabolic features.

**Figure 2:**
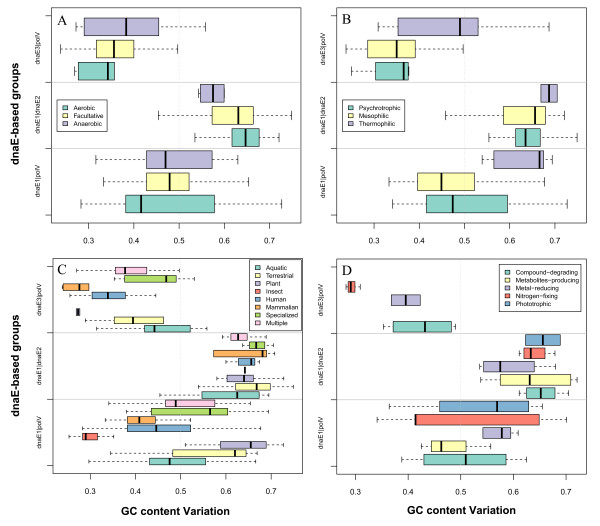
**Bacteriological features and GC content variations**. GC content variations across the three dnaE-based groups examined for different bacteriological features: oxygen requirement (A), thermal adaptation (B), habitat (C), and metabolic features (D).

First, we considered three different oxygen requirements--aerobic, facultative, and anaerobic--and examined them among 302 eubacteria in the three dnaE-based groups (Table 2). Among 124 aerobic bacteria, the dnaE1|dnaE2 (55.7%) and the dnaE1|polV (39.5%) are the major groups, not the dnaE3|polV (4.8%) group. The 64 anaerobic bacteria show a different distribution compared to the aerobic bacteria: the dnaE1|polV (57.8%) and dnaE3|polV (32.8%) groups are more abundant. The 114 facultative bacteria show a more balanced distribution between the dnaE1|polV (36.8%) and dnaE3|polV (39.5%) groups, but both are slightly more abundant than the dnaE1|dnaE2 group (23.7%). This result suggests that bacteria with the dnaE1|polV combination are more versatile or robust, as they show less oxygen constraint, collectively, than the other two groups. dnaE1|dnaE2 bacteria tend to be aerobic and facultative, and dnaE3|polV bacteria tend to be mostly anaerobic and facultative. Two-way ANOVA analysis showed significant GC differences among the dnaE-based groups (*F *= 153.7, *P *< 0.0001), but not among bacteria under different oxygen requirements (*F *= 0.160, *P *= 0.852). Thus, our analysis doesn't appear to support the oxygen requirement hypothesis (Table 1 and Figure 2A), and only among members of the dnaE1|dnaE2 group do aerobic bacteria have higher average GC contents than their anaerobic counterparts.

**Table 2:** Oxygen requirements of the dnaE-based groups

	Total	dnaE1|polV	dnaE1|dnaE2	dnaE3|polV
**Aerobic**	**124 **(100%)	49 (39.5%)	69 (55.7%)	6 (4.8%)
**Facultative**	**114 **(100%)	42 (36.8%)	27 (23.7%)	45 (39.5%)
**Anaerobic**	**64 **(100%)	37 (57.8%)	6 (9.4%)	21 (32.8%)
**Total**	**302**	**128**	**102**	**72**

Note: The number of bacteria is provided for each oxygen obligation class, and the corresponding percentages are given in parentheses. There is no precise oxygen requirement information for 62 of the bacteria in our collection.

**Figure 3:**
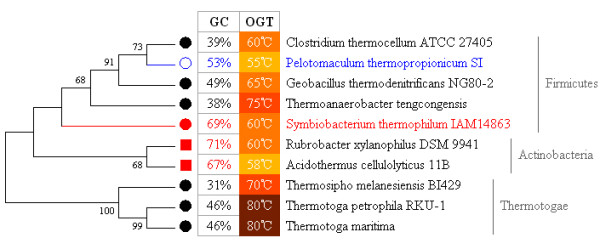
**Contribution of alpha-dimer asymmetry and optimal growth temperature (OGT) to GC content variation**. This dataset contains 10 thermophilic bacteria of three different phyla (five from Firmicutes, two from Actinobacteria, and three from Thermotogae), which have a broad GC content (from 31% to 71%) and OGT (from 55°C to 80°C) variation. The solid circles denote bacteria of the dnaE3|polV group, with two exceptions (one, labeled in blue, which lost *polC*, and the other, labeled in red, has an insertion of *dnaE2)*. The red squares denote bacteria of the dnaE1|dnaE2 group. The phylogenetic tree was constructed using the NJ method of MEGA 4.0 by using 16s rRNA sequences. Bootstrap values (>50) are labeled.

Second, we classified our data according to the optimal growth temperature (OGT, an indicator of thermal adaptation) (Table 3). Most of the thermophilic bacteria are classified into the dnaE3|polV group and most of the psychrotrophic bacteria are from either the dnaE1|polV or dnaE1|dnaE2 groups. Two-way ANOVA analyses demonstrated significant GC content variations among dnaE-based groups (*F *= 154.4, *P *< 0.0001) and among bacteria of different OGTs (*F *= 14.0, *P *< 0.0001). In all three dnaE-based groups, the data confirmed the thermal adaptation hypothesis (Table 1), where thermophilic bacteria tend to have a higher GC content than non-thermophilic bacteria (Figure 2B). Further correlation analysis confirmed a linear relationship between these two factors, despite the fact that mesophilic bacteria are more abundant, but the dnaE3|polV group bacteria showed stronger and more significant correlations (*R *= 0.437, *P *< 0.01; additional file 1). However, we should be cautious in interpreting this correlation; for example, two *Thermotoga *species with an average OGT of 80°C have a lower GC content (46%) than two *Actinobacteria *species (69%) with an average OGT of ~59°C (Figure 3). Bacteria are able to survive under both harsh and favorable environments, such that the sequence signatures of their genomes' compositional changes may not always be directly related to their bacteriological characteristics [33].

**Table 3:** Temperature adaptations of the dnaE-based groups

	Total	dnaE1|polV	dnaE1|dnaE2	dnaE3|polV
**Psychrotrophic**	**75** (100%)	34 (45.3%)	36 (48.0%)	5 (6.7%)
**Mesophilic**	**116 **(100%)	47 (40.5%)	31 (26.7%)	38 (32.8%)
**Thermophilic**	**21 **(100%)	6 (28.6%)	2 (9.5%)	13 (61.9%)
**Total**	**212**	**87**	**69**	**56**

Note: We classified bacteria as 'psychotropic' whose environmental temperature requirement < = 30°C, 'mesophilic' < = 45°C, and 'thermophilic' > = 75°C. The number of bacteria in each group is provided for each temperature obligation class, and their percentages in each class are given in parentheses.

Third, we investigated bacteria under different environments, such as habitat and host (Table 4). The analysis indicated that the dnaE1|polV bacteria are still the most broadly distributed and the other two groups of bacteria are relatively restricted as to their environments. The limited number of bacteria analyzed may have introduced a degree of bias; however, most terrestrial and plant-associated bacteria (67.9% and 64.0%) belong to the dnaE1|dnaE2 group, whereas most pathogenic or symbiotic bacteria in insects, humans, and mammals, as well as those dwelling in aquatic and other specialized environments, fall into the dnaE1|polV group. In addition, a significant proportion of dnaE3|polV bacteria (24/69) appeared to be specialized for human hosts. Only bacteria involved in multiple lifestyles exhibit no obvious distribution disparity among all three groups (Figure 2C). The terrestrially-dwelling bacteria have higher GC contents than aquatic-dwelling bacteria, according to the environment pressure hypothesis (Table 1), in both the dnaE1|polV and dnaE1|dnaE2 groups, but not in the dnaE3|polV group.

**Table 4:** Hosts of the dnaE-based bacterial groups

	Total	dnaE1|polV	dnaE1|dnaE2	dnaE3|polV
**Aquatic**	**76 **(100%)	42 (55.3%)	28 (36.8%)	6 (7.9%)
**Terrestrial**	**28 **(100%)	3 (10.7%)	19 (67.9%)	6 (21.4%)
**Plant**	**25 **(100%)	7 (28.0%)	16 (64.0%)	2 (8.0%)
**Insect**	**18 **(100%)	17 (94.4%)	1 (5.6%)	0 (0.0%)
**Human**	**69 **(100%)	38 (55.1%)	7 (10.1%)	24 (34.8%)
**Mammalian**	**42 **(100%)	26 (61.9%)	10 (23.8%)	6 (14.3%)
**Specialized**	**22 **(100%)	13 (59.1%)	3 (13.6%)	6 (27.3%)
**Multiple**	**63 **(100%)	19 (30.2%)	25 (39.7%)	19 (30.2%)
**Total**	**343**	**165**	**109**	**69**

Note: The number of bacteria is provided for each habitat in each group. The percentage of bacterial species in each category is given in parentheses. 13 bacteria have no available habitat information and eight bacterial habitats in other unlisted hosts are not included (two in avians, two in bivalves, one in earthworms, one in nematodes and two in Pisces).

Fourth, we correlated metabolic activity with GC content and our dnaE-based grouping scheme. Interestingly, the majority of compound-degrading, metabolite-producing, and nitrogen-fixing bacteria tend to be members of the dnaE1|dnaE2 group, whose GC content is always higher than the other two groups (Table 5; Figure 2D).

**Table 5:** Metabolic features of eubacteria in the dnaE-based groups

	Total	dnaE1|polV	dnaE1|dnaE2	dnaE3|polV
**Compound-degrading**	**29 **(100%)	4 (13.8%)	21 (72.4%)	4 (13.8%)
**Metabolites-producing**	**10 **(100%)	3 (30.0%)	7 (70.0%)	0 (0.0%)
**Metal-reducing**	**11 **(100%)	5 (45.4%)	4 (36.4%)	2 (18.2%)
**Nitrogen-fixing**	**18 **(100%)	5 (27.8%)	8 (44.4%)	5 (27.8%)
**Phototrophic**	**10 **(100%)	8 (80.0%)	2 (20.0%)	0 (0.0%)
**Total**	**78**	**25**	**42**	**11**

Note: The number of bacteria is provided for each metabolic feature in each group. The percentage of bacterial species in each category is given in parentheses.

### The correlation of mutator genes, *dnaE2 *and *polC*, to GC content variation

**Figure 4:**
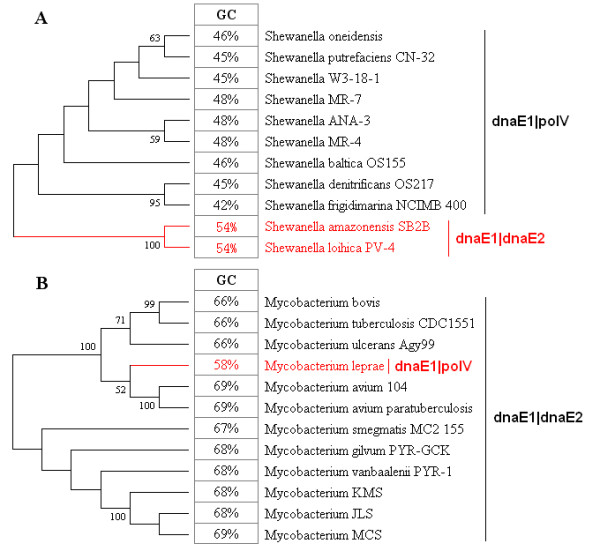
**Correlation between dnaE2 gain-and-loss and GC content variation**. In the Genus *Shewanella*, two bacteria (red) have higher GC content, which correlates well with the presence of the *dnaE2 *gene (A). *Mycobacterium leprae *has a lower GC content because it has lost the *dnaE2 *gene (B). The phylogenetic tree was constructed using the NJ method of MEGA 4.0 and 16s rRNA sequences. Bootstrap values (>50) are labeled.

The two mutator genes, *dnaE2 *and *polC*, alter the GC content in different ways. To correlate *dnaE2 *to GC content variation, we examined two specific genera, *Shewanella *of the phylum y-Proteobacterium and *Mycobacterium *of the phylum Firmicutes (Figure 4A and 4B, respectively). Even within the same genus (Figure 4A), the bacteria possessing *dnaE2 *(dnaE1|dnaE2 group) have a higher genomic GC content (54%) than those that do not possess *dnaE2 *(dnaE1|polV, showing an average GC content of 46%). Similarly, we found that *M. leprae *(genus *Mycobacterium) *has a decreased GC content (by about 10%) that correlates with its loss of *dnaE2 *(Figure 4B). With regard to the dnaE3|polV group, only one bacterium, *Pelotomaculum thermopropionicum SI*, has lost *polC *[34], and has consequently acquired a relatively high GC content (53%) compared to the average of other group members (about 42%). One bacterium, *Symbiobacterium thermophilum IAM 14863 *of the Firmicutes, was predicted to have a low GC content similar to other bacteria of the same phylum; however, its GC content was 69%, and further thorough genomic sequence screening showed that it contains an extra copy of the *dnaE2 *gene (Figure 3).

### The correlation of genome size with GC content

**Figure 5:**
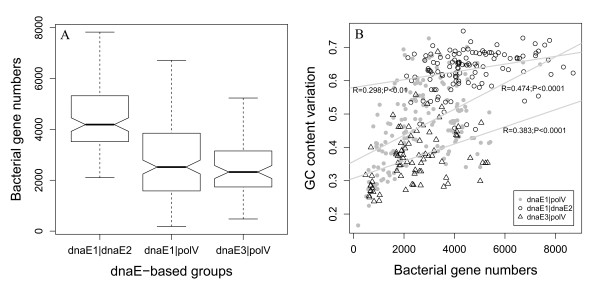
**Gene numbers across the dnaE-based groups**. The box-plots show gene numbers (A) and the correlation between gene number and GC content variation (B) across the dnaE-based groups.

To investigate the relationship between genome size and GC content, gene number was plotted (gene number and genome size are correlated linearly [35]) against genomic GC content (Figure 5). The graph indicates that bacteria of the high-GC content group-- dnaE1|dnaE2--have relative larger genomes, on average, than those of the other two groups (Figure 5A). GC content and bacterial genome size correlate positively and significantly in both the dnaE1|polV (*R *= 0.474, *P *< 0.0001) and dnaE3|polV (*R *= 0.383, *P *< 0.0001) groups. However, it is less obvious in the dnaE1|dnaE2 group (*R *= 0.298, *P *< 0.01; Figure 5B).

**Figure 6:**
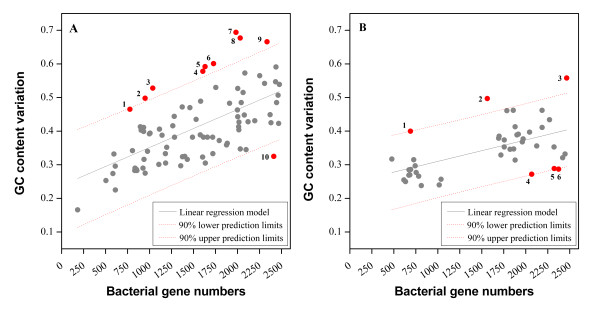
**Linear correlation between genome size and GC content**. Bacterial genome sizes are represented by the number of genes. Bacteria with less than 2,500 genes were chosen for analysis in dnaE1|polV (A) and dnaE3|polV (B), respectively. There is a strong and significant correlation between genome size and GC content after eliminating outliers (red solid circles). *R *values change from 0.6179 to 0.7479 (p < 0.0001) in the dnaE1|polV group and from 0.5571 to 0.8172 (p < 0.0001) in the dnaE3|polV group. The linear model is *Y *= *0.0001128X + 0.2387 *for the dnaE1|polV group and *Y *= *0.00006374X + 0.2464 *for the dnaE3|polV group. 90% upper and lower prediction limits are also shown. All the numbered outliers were further analyzed to interpret potential reasons underlying this correlation (Table 6).

Considering a specific range of gene numbers, such as less than 2,500 genes, we found that the correlation between GC content and gene number increases considerably; the dnaE1|polV and dnaE3|polV bacteria have *R *values 0.6179 (*P *< 0.0001) and 0.5571 (*P *< 0.0001), respectively (Figure 6A and 6B). We did not include the dnaE1|dnaE2 bacteria in this analysis, because their genomes tend to be much larger with an average of 4,587 genes (only six bacteria have 2,000 to 2500 genes). Stronger significant correlations were observed after the outliers were eliminated: *R *values changed from 0.6179 to 0.7479 (*P *< 0.0001) in the dnaE1|polV group (Figure 6A) and from 0.5571 to 0.8172 (*P *< 0.0001) in the dnaE3|polV group (Figure 6B). In addition, we found that the dnaE1|polV group has a steeper slope than that of the dnaE3|polV group.

The dnaE3|polV group, whilst obeying the overall correlated trend, behaves in a distinct way (Figure 6B); bacteria with smaller genome sizes (less than ~1,500 genes) have a slightly decreased genomic GC content coupled with an increase in gene number. This decrease is also seen in the genomes with more than ~1,500 genes, but to a lesser extent. In other words, the GC content correlation of this bacterial group should be reexamined when more data becomes available. To resolve the issue of whether other mutator genes are involved in causing outliers from the rule, we further analyzed all 16 outliers. The three most frequently detected mutator genes in these bacteria were the GC-increasing *mutT*, the AT-increasing *mutY*, and the role-to-be-defined *mutM *genes (Table 6). The nine outliers (shown in Figure 6A) of the dnaE1|polV group categorized as higher GC were all confirmed to have lost their *mutT *gene, and one bacterium categorized as higher AT has lost its *mutM *gene, based on tBLASTn analysis. Similarly, among the dnaE3|polV group (Figure 6B), three high GC bacteria have lost their *mutT *gene and three high AT bacteria do not possess *mutM*.

**Table 6:** Mutator genes and GC content variations in the dnaE-based groups

dnaE-based Groups		**No**.	Bacteria	mutT	mutY	mutM
		1	Tropheryma whipplei TW08 27	-	-	+
		2	Anaplasma marginale St Maries	-	+	+
		3	Treponema pallidum	-	+	-
		4	Bifidobacterium longum	-	+	-
dnaE1|polV	High-GC	5	Bifidobacterium adolescentis ATCC 15703	-	+	-
		6	Mycobacterium leprae	-	+	+
		7	Thermus thermophilus HB27	-	+	+
		8	Leifsonia xyli xyli CTCB0	-	+	+
		9	Deinococcus geothermalis DSM 11300	-	+	+
	
	High-AT	10	Flavobacterium psychrophilum JIP02 86	+	+	-

		1	Mycoplasma pneumoniae	-	-	+
	High-GC	2	Lactobacillus delbrueckii bulgaricus	-	-	+
dnaE3|polV		3	Moorella thermoacetica ATCC 39073	-	+	+
	
		4	Fusobacterium nucleatum	+	+	-
	High- AT	5	Clostridium novyi NT	+	+	-
		6	Clostridium tetani E88	+	+	-

Note: Bacteria are numbered as in Figure 6. In the dnaE1|polV group, there are 10 outliers; however, nine belong to the high-GC category and only one to the high-AT category. In the dnaE3|polV group, three belong to the high-GC and three to the high AT-category. "+" and "-"represent the presence and absence of mutator genes, respectively.

## Discussion

### The gain-and-loss of mutator genes underlies GC content variation

Deficiencies in mutator genes can dramatically increase the mutation rate [31,32,36,37]. For example, in the absence of both *mutY *and *mutM*, thousands-fold increase in CG-to-AT mutations was observed, and the same magnitude of mutations is evident in *mutT*-deficient strains, but with an opposite mutation spectrum, namely AT-to-GC [37]. Therefore, the isolation and characterization of mutator genes have led to a better understanding of mutation mechanisms. Mutation-driven bacterial adaptive strategies to the environment are widely reported to be beneficial for bacteria in surviving periods of stress, such as starvation and drug exposure [37-40]. It could be argued that such mutator loss is very rare in evolution, yet there is evidence indicating that the incidence of mutator strains among pathogenic isolates is quite high [37,41].

### The mutators, *dnaE2 *and *polC*, are two major contributors to GC content variation

Our analysis demonstrates that the existence of *dnaE2 *an*d polC *is associated with higher GC (>50%) and lower GC contents (<50%), respectively. To further verify the association between dnaE dimer asymmetry and GC content variation, we also carried out two case studies on several closely related bacteria to exclude the contribution of phylogenetic distance, because GC content also displays a strong phylogenetic signal [42]. Our results clearly indicated that gain-and-loss of *dnaE2 *can greatly increase or decrease the GC content, respectively, providing further evidence that *dnaE2 *is the major contributor to GC content variation in the dnaE1|dnaE2 group. In addition, we also found that a single copy of *dnaE2 *in *S. thermophilum IAM 14863 *leads to an Actinobacteria-like high GC content. There has been some debate about the status of this bacterium: whether it belongs to the Actinobacteria because of its high GC (69%) or to Firmicutes, which share its bacteriological features. Recently, it was confirmed that *S. thermophilum IAM 14863 *is a member of the Firmicutes [43], and our analysis agrees with that study and suggests that its Actinobacteria-like high GC content is a result of an additional copy of *dnaE2*, possibly gained through horizontal gene transfer (HGT). Its higher GC content should not be considered as a factor confounding its taxonomic position. Furthermore, increasing evidence indicates that *dnaE2 *may participate in SOS mutagenesis through the TLS pathway instead of replication [44-47], as it is a possible member of the error-prone Y family polymerases. Furthermore, bacteria without *dnaE2 *normally have the TLS-related *polV *for functional compensation [48,49]; therefore, we believe that these polymerases are associated with the replication machinery and have strong influences on DNA synthesis, leading to biased compositional changes (e.g., pol η and pol κ lead to AT-rich DNA and pol ζ and Rev1 lead to GC-rich DNA) [41].

As to the relationship between *polC *and high AT content, we only found one example, namely bacterium *P. thermopropionicum SI*, whose loss of *polC *is consistent with its higher GC content as compared to the average of other Firmicutes. In addition, we found that the linear correlation between GC content and genome size in the dnaE3|polV bacteria tends to have a less steep slope compared with that in the dnaE1|polV group, which further suggests that *polC *may be responsible for the lower level of GC content in the dnaE3|polV group.

### The loss of AT-increasing mutator genes may contribute to genome size reduction and GC content variation

Our analysis showed that *Treponema pallidum *(#3) has lost *mutT *but possesses *mutY*. The lost of *mutT *may be related to its 15% higher GC content as compared to its phylogenetically closely related relative, *T. denticola ATCC 35405*, which has both *mutT *and *mutY*. A similar situation is also found in *Anaplasma marginale St Maries *(#2). However, the reason it has a higher GC content (8%) than the closely related *A. phagocytophilum HZ *is not because of its loss of *mutT*, as neither of them possess *mutT*, but may be attributable to the absence of *mutY *in the latter bacterium. Despite the fact that dnaE1|dnaE2 bacteria were not included in this part of the analysis, we still managed to find an example. Yoji Nakamura et al. found that the GC content of *Corynebacterium efficiens *is 10% higher than that of *C. glutamicum *and *C. diphtheriae*, probably because it lacks *mutT *[50]. Whether each mutator gene is a causative factor for a particular GC content variation requires further experiments and a larger dataset, which may prove problematic when HGT is factored in.

It is well established that genome size is positively correlated with GC content. Our analyses not only confirmed this notion, but also showed that this correlation is more pronounced in the dnaE1|polV and dnaE3|polV groups, especially when the gene number of each bacterium is less than 2,500. Generally, bacteria with <2,500 genes often experience genome reduction or gene loss. Therefore, the strong and significant positive correlation between genome reduction and AT increase may reflect dramatic gene losses, especially the loss of mutator genes, because mutator gene defects cause AT increase more than GC increase [37] (Additional file 2). To test this hypothesis, the correlation between GC content and gene number for bacteria possessing less than 2,500 genes was examined, revealing the underlying reasons for these outliers. For instance, those belonging to 'high-GC' are all confirmed to have lost their *mutT *gene. In other words, when a genome suffers a significant size reduction, it most likely experiences both loss of mutator gene (s) and AT-increase. The fact that most insect pathogens undergo genome reduction and possess AT-rich genomes is testimony to this hypothesis [51-53]. A more rigorous analysis is required to confirm whether the observed higher number of *de novo *GC-AT mutations [54,55] are directly related to the loss of AT-increasing mutator genes.

A recent study investigated specificity and rates of different mutational biases of the *Salmonella typhimurium *genome in the absence of major DNA repair systems [56], where mutator genes result in GC-to-AT mutations. By sequencing two *S. typhimurium *mutants grown for 5,000 generations, they observed that the mutation spectrum coincides with the expected pattern, where among the 943 identified nucleotide substitutions, 91% were GC-to-TA transversions and 7% were GC-to-AT transitions [56]. This is the first large-scale genomic level experiment that confirms the relationship between mutator genes and genome GC variation, and strongly supports our hypothesis.

### Environmental factors do correlate with GC variation, but to a variable extent

Our dnaE-based grouping scheme not only guides GC content analysis, but also provides a framework for the analysis of different environmental factors. Taking temperature as an example, we found that thermophilic *Thermoanaerobacter tengcongenesis*, presumed to have a higher GC content, and non-thermophilic *Streptomyces coelicolor*, presumed to have a lower GC content, actually have genomic GC contents of 38% and 72%, respectively. However, our grouping scheme explains the contradiction: the former is a dnaE3|polV bacterium, while the latter is a dnaE1|dnaE2 bacterium.

Another minor correlation between GC content and environmental factors was found when the habitats of various bacteria were examined. It was reported that the environment plays an active role in shaping GC content, such as surface water vs. soil, and indeed, bacteria living in aquatic conditions have an average GC content of ~34%, whereas soil-dwellers have an elevated GC content of ~61% [26]. Our grouping scheme confirms that the former are mostly dnaE1|polV bacteria and the latter are mostly dnaE1|dnaE2 bacteria. But the six aquatic bacteria are observed to have higher GC content than soil-dwelling bacteria within the dnaE3|polV group. Further analysis reveals that, among the six aquatic bacteria analyzed, five are thermophiles and one is uranium/chromium-reducing. This also raises the question as to whether dwelling conditions are relevant or if they are simply an ascertainment bias introduced by the difference of species distribution under different environmental conditions or metagenomics. Therefore, we should be very cautious when addressing the relationship between environmental or bacteriological features and genomic GC content, especially when the number of genomes analyzed is rather limited.

In summary, although the contribution of oxygen requirement, nitrogen-fixing, terrestrial dwelling, and larger genome size to GC content variation has been discussed within a unified scheme, some of the previously identified correlations (Table 1) should be reconsidered, as there is a higher chance for these bacteria to be members of the dnaE1|dnaE2 group. Therefore, taxonomy-based classification should be factored in for this type of analyses when there are sufficient sequenced genomes in the near future.

### Is GC content variation intrinsic or driven by environmental factors?

Based on our dnaE-based grouping scheme, we believe that GC content variation is governed by replication and repair mechanisms, but is influenced by environmental factors. As prokaryotes, eubacteria are robust, but have never evolved to be more complex. Such robustness builds upon genome variations that are promulgated by a large population. These variations in genome compositions permit the loss, acquisition, or change in DNA sequences. When such composition dynamics are at work, bacterial GC contents comply with our grouping scheme, regardless of whether they are mutating for the better or are being selected and suffering a bottleneck. For detailed tendencies, specific conditions should be investigated and different mechanisms proposed. Future investigations will comprise more detailed analysis of outliers that either have extreme GC contents, or do not follow the dnaE-based rules. Experiments to construct new organisms whose grouping scheme is disrupted will also be performed. Extreme environmental conditions could be applied to the three bacterial groups separately to enforce selective pressure to determine if they are able to produce the predicted mutation spectrum mirroring that seen in naturally isolated counterparts.

## Conclusion

DNA polymerase III α subunit and its isoforms participate either in replication (such as *polC) *or in SOS mutagenesis/TLS (such as *dnaE2*), playing a dominant role in producing GC variations that can be classified into three basic spectra: GC variable, high GC, and low GC groups. Mutator genes, especially those that have dominant effects on mutation spectra towards either GC or AT content biases, can also alter GC content in either direction to a certain extent. For example, the presence of *dnaE2 *is a definite sign of higher GC content. Increased bacterial genome size (gene number) appears to rely on genomic GC content increase. However, it is unclear whether the changes are directly related to certain environmental requirements. Indeed, environmental factors do influence GC content variation, but the correlations are more obvious when analyzed under our dnaE-based grouping scheme. For example, most terrestrial, plant-associated, and nitrogen-fixing bacteria are of the dnaE1|dnaE2 group, whereas most pathogenic or symbiotic bacteria in insects, and those live in aquatic environments, belong to the dnaE1|polV group.

## Methods

### Genomic data

The non-redundant eubacterial grouping was based on a random selection of a single isolate or strain from the collection in the NCBI (National Center for Biotechnology Information) databases (ftp://ftp.ncbi.nih.gov/genomes/Bacteria/), yielding 364 non-redundant bacterial genomes. We classified them into dnaE1-dnaE1|polV (173 genomes), dnaE1-dnaE1|dnaE2 (115 genomes), and polC-dnaE3|polV (76 genomes) according to their presence of DNA polymerase III alpha subunit and damage-inducible *dnaE2 *or *polV.*

### Bacteriological information

We collected most of the related information for the 364 non-redundant bacterial dataset from the Bergey's Manual of Determinative Bacteriology (9th edition, 1994) [57], NCBI's Entrez Genome Project database (http://www.ncbi.nlm.nih.gov/sites/entrez?db=genomeprj).

To avoid the interference of phylogenetic distance with GC content, we selected two special groups, *Shewanella *and *Mycobacterium*, where there are sufficient closely related genomes for the analysis, yet they belong to two different dnaE-based groups, dnaE1|polV and dnaE1|dnaE2, respectively. In addition, we constructed an OGT dataset to analyze the relationship between OGT and GC content. We randomly chose ten thermophiles with definite OGTs across three phyla (Firmicutes, Actinobacteria, and Thermotogae) and in two dnaE-based groups (dnaE1|dnaE2 and dnaE3|polV) for an in-depth analysis. We employed MEGA (version 4.0) [58] to construct all phylogenetic bootstrap trees using the neighbor-joining method [59] based on 16S rRNA sequences.

### Identification of mutator genes

To identify mutator genes, we collected 13 experimentally confirmed common mutator genes and used the online BLAST tools (http://blast.ncbi.nlm.nih.gov/Blast.cgi) for *in silico *identification in all candidate bacterial genomes. Both protein size (the cutoff value > 50%) and sequence homology (E-value 1 × 10^-5^) were considered.

## Competing interests

The authors declare that they have no competing interests.

## Authors' contributions

HW carried out sequence analysis, biological information classification, and drafted the manuscript. ZZ, SNH, and JY designed and supervised the project and revised the manuscript. All authors read and approved the final manuscript.

## Reviewers' comments

### Reviewer 1

Nicolas Galtier, CNRS-Université Montpellier II, France

This article revisits the literature about genomic GC-content distribution across bacteria in the light of variations in the structure of the catalytic subunit of DNA polymerase III. Three classes of the dimeric subunit of DNA pol III have been described in bacteria, each influencing the genomic GC-content in a specific way.

This paper confirms/demonstrates that DNA pol III is a major determinant of between-species GC-content variations in bacteria, and pinpoints a couple of previous studies in which inappropriate conclusions were reached by not accounting for this effect.

In my opinion, this manuscript contains two important results, which revive and illuminate long-lasting controversies. The first one is about the relationship between GC-content and aerobiosis. We have known for ten years or so that aerobic bacteria show a higher GC-content than anaerobic ones, on average, and this is paradoxical given that C->T and G->A are generally the most common mutations in oxidative context. This study demonstrates that the relationship is largely, or entirely, explained by the differential usage of DNA pol III subunit between aerobes and anaerobes: aerobes tend to carry the GC-enriching polymerase, and anaerobes the AT-enriching one. The second strong result, in my opinion, is about the relationship between genomic GC-content and optimal growth temperature (OGT), two variables that were found unrelated across prokaryotes [60,61]. Here it is shown that, within each of the three categories of DNA pol III, GC% and OGT do correlate positively. The reason why this relationship did not come out in all-species analyses is that thermophiles most frequently use the AT-enriching polymerase, and mesophiles or psychrophiles the GC-enriching one. It seems to me that these two results, if confirmed, should have a strong impact on bacterial comparative and environmental genomics, in which GC-content variations are obvious, and so far poorly understood.

That said, I have a number of comments/concerns about the form of the paper, the underlying statistics, and its potential implications, which I hope might help improve the manuscript.

Paper organization

- I would suggest introducing the current work as an attempt to account for a confounding factor so far overlooked. Currently the manuscript focuses on their importance of replication genes in GC-content variations, but this very result was previously published (by the same authors), and this study does not add so much to that argument.

***Authors' response**: We appreciate the reviewer's encouragement and suggestions. We have restructured our manuscript to emphasize the correlations between relevant confounding factors and GC content variation. In this study, we found several lines of solid evidence, which confirmed our previous conclusions, based on large-scale comparative genome analyses*.

- Rather, I would suggest developing the two results I outline above: specifically review the relevant bibliography; show the GC%/OGT relationship within DNA pol groups, and globally (similarly to figure 5b); perform two-way ANOVA of GC% on DNA pol category and OGT (on one hand), and on DNA pol category and aerobiosis (on the other hand), and discuss the percentage of variance of GC% explained by these variables; conclude about misinterpretations in existing literature.

***Authors' response**: We agree. We re-analyzed the relationship between GC% and OGT (see additional file *1*) and have added a new reference referring to a relevant result from one of our early studies. We also performed the corresponding two-way ANOVA analyses and incorporated the results into the revised manuscript*.

- By comparison, it seems to me that the analyses of ecological and metabolic features and of genomic gene content (figure 2c, 2d, 5, 6) add less to existing bibliography. I would suggest shortening these sections, and especially the section about gene number, in which separating species by DNA pol III classes does not appear to change much of the prevailing hypotheses.

***Authors' response**: After analyzing the contribution of OGT and oxygen requirement to GC content variation, based on our dnaE-based group framework, we think that it is necessary for us to perform analysis on the contribution of other related factors, such as several ecological and metabolic features, to provide evidence for the universality of the dnaE-based grouping scheme. For example, plant- and terrestrial-associated bacteria that are reported to have higher GC content are mostly grouped in the dnaE1|dnaE2 group. Therefore, we think that some of the previously described relationships between GC content and environmental factors may also fall into our scheme, but have not been realized. Indeed, from Tables *4* and *5*, we observe that there are still not enough data for a meaningful statistical analysis. We hope that we can draw a more significant conclusion in the near future, when more bacterial genome sequences become available. As to the analysis performed on gene number, our major conclusion is that the dnaE2 group bacteria that have a higher GC content tend to have larger genomes, in contrast to the opposite situation in the dnaE3 group bacteria. Therefore, we believe that the positive correlation between genome size (or gene number) and GC content is much more pronounced when analyzed under our dnaE-based grouping scheme*.

Statistical issues

- The manuscript does not explicitly address the problem of phylogenetic independence of the observations. The author might think of using the Independent Contrast method, or any related method, to check further the significance of the relationships they uncover. At any rate, the authors must give an idea of the phylogenetic distribution of the three classes of DNA pol III: are they scattered throughout the bacterial tree, or clustered by phyla/families? This is partly answered by figure 4, in which within-genus variations of DNA pol III class are reported, somewhat suggesting that the phylogenetic inertia on this trait is weak. Confirmation welcome.

***Authors' response**: We fully agree with the reviewer and it would be compelling to analyze the phylogenetic independence of these observations. However, it is not straightforward to illustrate these points in the current manuscript and we believe that it is beyond the scope of this manuscript. We have prepared another manuscript on the evolutionary scenarios of these four different polymerases, as well as analysis of their relationship in a context of both bacterial taxonomy and sequence evolution*.

- Figure 3, figure 4 and many sentences in the manuscript make convincing cases suggesting that changes in DNA pol III affect bacterial GC-content evolution. However, I wonder how representative are these examples: were they specifically selected to illustrate the main pattern reported in this study, or are they more or less random instances? Figure 3: why choosing just ten thermophilic species, and why these ten?

***Authors' response**: We thank the reviewer for his constructive comments. We wanted to explain the ambiguous relationship between OGT and GC content based on real data. The reasons we choose these 10 bacteria are as follows. First, we needed to select bacteria that have precise OGT information. Second, to exclude the interference of phylogenetic distance with GC content, we need to select several bacteria that have close phylogenetic relationships in each phylum. Third, all the bacteria should fall into the three different dnaE-based groups evenly. Fourth, both their GC content and OGT have to vary significantly*.

Figure 4: are Shewanella and Mycobacterium the only genera showing variations in DNA pol III? If not, could you please provide a more global picture, and mention counter-examples if there are some? I have a similar concern about the discussion, in which the focus is presumably put on examples fitting the general theory, not counter-examples.

***Authors' response**: We analyzed a collection of what are currently available in the public domain and have not found a single example that contradicts our grouping scheme and predictions concerning the trend of GC content variation in relationship with other extrinsic factors. Our large-scale comparative screening demonstrated that most closely related bacteria tend to have the same isoforms of dnaE polymerases. We also identified two examples, namely, Shewanella and Mycobacterium, where the rules are not followed but the explanation is apparent*.

- Along the same lines, the removal of "outliers" (figure 6) does not appear justified to me, even though I agree that horizontal gene transfer presumably perturb the observed relationship, which is good to mention.

***Authors' response**: Agreed. We further revised the corresponding description by performing linear regression analysis and removing the "outliers" by more robust upper and lower 90% prediction limits*.

Implications

- It seems to me that the surprising report by Foerstner et al. [26] of very different GC- content distributions between distinct environmental samples (despite comparable representation of the bacterial phyla) could reflect a differential usage of the three DNA pol III across environments. This could perhaps be checked by identifying DNA pol III sequences in the corresponding metagenomic data.

***Authors' response**: You are right. We also think that DNA polymerase III may be an excellent group of genes for phylogeny and related evolutionary analysis. We are currently working on several metagenomic data and will apply this idea and report the results as soon as we have concrete conclusions*.

- Having demonstrated that the DNA pol III subunit plays a major role in GC% variations, it is tempting to ask what determines variations in DNA pol III usage across groups of bacteria. For instance: do aerobic bacteria most frequently use the GC-enriching DNA pol III because it is GC-enriching, or because it is more efficient in aerobic conditions, and incidentally GC-enriching?

***Authors' response**: The reviewer poses a very interesting and challenging question here. We believe that the four dnaE isoforms diverged at a very early stage of eubacterial evolution and drove the bacteria towards not only different GC contents, but also different evolutionary routes or landscapes, either randomly or under environmental pressures. Over time, bacteria that possess different dnaE isoforms have favored different environments, leading to the current diversity*.

Form

- The manuscript would strongly benefit from English corrections

- Abstract (and introduction, last paragraph):

"The contribution of other environmental or bacteriological factors, such as genome size, temperature, oxygen requirements, and habitats, either indirectly rely on the choice of mutator genes or take the advantage of their fine-tuning effect on the trends determined by other factors." This sentence is unclear to me and probably deserves rephrasing.

***Authors' response**: We have rephrased this paragraph*.

- The Background section introduces codon usage biases and transcription-coupled mutation/repair, but these two aspects are not addressed in this study. The potential role of OGT, aerobiosis, metabolism and environment are not, or very briefly, introduced.

***Authors' response**: Our previous study confirmed that codon usage biases are driven by GC content changes, but not vice versa *[9]*, as suggested by Knight et al. *[8]*. Therefore, we did not pay too much attention to this point here. The contribution of transcription-coupled repair was discussed in Gramineae *[10]*, but we are still uncertain how to analyze this in bacteria. For the convenience of the discussion, we summarized 10 other different hypotheses that have been put forward as potential mechanisms for generating GC content variation (Table *1*), and we will write a more comprehensive review when the conclusions become clearer*.

- Table 2 and figure 2a: I suggest grouping "microaerophilic" with "anaerobic" (or "microaerophilic" with "facultative" if you think it is more appropriate). This is because percentages are meaningless in small groups of species, and percentages are very important in this table.

- Table 3 and figure 2b: I suggest grouping psychrophile with psychrotrophic bacteria, and thermophiles with hyperthermophiles (same reason).

***Authors' response**: Agreed. We have revised this in related tables and figures*.

- Figure 2a and 2b: keep the same order as in table 2 and table 3, respectively, for categories. ***Authors' response: ****We have the made revisions.*

### Reviewer 2

*Adam Eyre-Walker, Centre for the Study of Evolution and School of Life Sciences, University of Sussex, Brighton, United Kingdom*.

The current paper follows up work the authors have done on the relationship between genomic GC and the presence of various DNA polymerase alpha subunits in eubacterial genomes. They confirm, as in their previous work [28] that species which use a combination of dnaE3 and polC subunits tend to have lower genomic GC contents than those which use dnaE1 subunits, which have much lower genomic GC contents than those which use a combination of dnaE1 and dnaE2. They argue therefore that mutation biases introduced by the alpha polymerase is a major determinant of genomic GC content in bacteria.

Unfortunately, this conclusion is not justified given that there is a high level of phylogenetic non-independence in their data. If we accept their classification of alpha subunits into the four main familes (dnaE1-3 and polC) then almost all bacteria that have dnaE3 and polC are firmicutes and almost all bacteria with dnaE1 and dnaE2 bacteria are proteobacteria and actinobacteria [29]. Hence it is possible that the association between alpha polymerase subunits and GC content is coincidental, established by a few coincidental evolutionary changes; for example, it might be that the evolution of the dnaE2 subunit happened at the same as another unrelated evolutionary change which caused a shift towards high genomic GC content. If there have been relatively few instances in which the alpha polymerase has evolved then association with GC content may be coincidental.

***Authors' response**: We thank the reviewer for the critical comments. We have overlooked the molecular mechanisms that govern compositional (sequence) variations, but concentrated on sequence variation itself. A minute change in the conformation of these mutator enzymes may alter the GC content in another direction. Clearly, Figure *4* shows that in genera Shewanella and Mycobacterium, bacteria in the dnaE1|dnaE2 group generally have higher GC content (by about 10%) as compared with those in the dnaE1|polV group. In addition, we found that all three newly sequenced (deposited in the public database) bacteria in Firmicutes (the dnaE3 group) have unexpectedly high GC content (>60%) and two of them (Alicyclobacillus acidocaldarius subsp. Acidocaldarius DSM 446 and Symbiobactrium thermophilum IAM 14863) correlate well with the presence of dnaE2. One bacterium (Candidatus desulforudis audaxviator MP104C) has been proven to have lost polC, similar to what we found in Pelotomaculum thermopropionicum SI. Furthermore, analyzing the pattern and distribution of bacterial SSR (simple sequence repeats), we found one bacterium, Acidiphilium cryptum JF-5, which was previously identified as dnaE1|polV group bacterium, has now been proven to have SSR patterns similar to that of dnaE1|dnaE2 group bacteria. Our further genome-wide screening led to the discovery of a single copy dnaE2 in one of its plasmids (manuscript in preparation). Therefore, we think that the correlation between dnaE polymerases and GC content is a rule rather than coincidental and exceptional, albeit lacking direct experimental confirmation. Of course, we do not think that there are no exceptions to the rule, but we predict that they are the minority*.

The authors need to conduct a proper comparative analysis by, for example, selecting related pairs of bacteria that differ in their alpha-polymerase subunits. They give some examples at the end of the current paper, but they need to find more examples, and to find these without reference to the genomic GC content. Once they have set the problem within a proper comparative framework they can start to investigate the relative correlation between GC content and alpha polymerase subunits, genome size, lifestyle....etc.

***Authors' response**: We have conducted a comparative analysis by selecting bacteria that differ in their alpha-polymerase subunits, as shown in Figure *4*. In future investigations, we may be able to show more examples, but what we have now is limited by the availability of the relevant public data*.

As it stands I do not think there is much evidence to support the authors' hypothesis that GC content evolution is determined by alpha polymerase subunits. Even if this was proven it is evident from their figure 1 that a large proportion of the variance in genomic GC content is not explained by subunits, since there is a large variance in genomic GC content within each subunit category.

***Authors' response**: We cited our previous related papers and added several lines of evidence to support our hypothesis. It is true that GC content variation in each group varies to different extents. What we are emphasizing here are two concepts. One is the fact that there are boundaries or specific spectra in compositional variability. The dnaE1|polV group is the extreme, which appears to have no limit in GC content variation but is regulated by mutator genes. Other groups have boundaries and they either prefer low-GC or high-GC contents. The other concept is why GC content varies and the complexity required to explain such variability. Large variances within each subunit category reflect the complexity of diverse factors contributing to GC content variation. As exemplified in our manuscript, there are also many other mutator genes (such as mutT, mutY, and mutM), as well as several environmental and bacteriological factors contributing to GC content variations. Horizontal gene transfer is another major factor that often results in broader GC content variability; not only as a mechanism of genetic material exchange, but also the material itself often makes significant contributions*.

Quality of written English: Needs some language corrections before being published.

***Authors' response**: We have carefully checked the wording throughout the manuscript and revised the manuscript for clarity*.

### Reviewer 3

*Eugene Koonin, National Center for Biotechnology Information, NIH, Bethesda, Maryland, United States*.

Wu et al. claim to have solved a very old enigma, that of the molecular basis of the GC-content variation in bacteria. They come to the conclusion that the defining factor is the asymmetry of the DNA polymerase III dimer, in particular, the presence of one of the two mutator forms, polC or dnaE2. It is certainly plausible that the structure of the replicative DNA polymerase substantially contributes to mutational biases. Nevertheless, unfortunately, the data presented in the article do not convince me at all that the structure of polymerase III alone determines the GC-content or even contributes to it significantly. Part of the problem is the puzzling lack of statistical analysis in the paper: the authors simply report some base composition preferences in different groups of bacteria without presenting correlation coefficients let alone p-values. More importantly, I think the authors fail to recognize and properly interpret the current status of the study of evolution of nucleotide composition in bacteria and archaea (their references 54-56). By now it appears certain that there is mutational bias toward AT in all prokaryotes, and accordingly, the high GC-content seen in many bacteria and archaea is most likely due to selection pressure. Both the molecular mechanisms underlying the mutational bias and especially the selective factors that offset this bias are of major interest but I am afraid the current article does not significantly contribute to our understanding of this evolutionary conundrum.

***Authors' response**: We are grateful for the reviewer's critical comments. The conclusion we draw in this study is based on comparative analysis of genomic sequences and correlations between GC content and various bacteriological features are examined. We plan to design experiments to test our hypothesis by investigating mutation patterns in reporter genes or even on a genome-wide level after introduction or elimination of dnaE2. We hope that we can provide more convincing experimental evidence to answer this question in the near future*.

Quality of written English: Needs some language corrections before being published.

***Authors' response**: We have carefully proofread the manuscript and invited a native English-speaking colleague to edit our revised manuscript*.

### Final reports

**Reviewer 1**: I am still concerned by many of the methodological and conceptual problems raised by the reviewers, which were only partially addressed in this revised version, in my opinion.

***Authors' response**: This is a fair assessment. We apologize for not be able to meet all expectations from the reviews. It is a spirit of scientific research that a publication should not easily satisfy a scientific question in a one-on-one fashion but stimulates deeper thinking and generates even more questions. Nevertheless, we will try to address some of the legitimate concerns in our future work*.

**Reviewer 3**: Unfortunately, the authors do not address the substance of the criticisms in their responses to reviewers. Neither have they made adequate language corrections.

***Authors' response**: We have added more analysis to the first revision and addressed some of the questions raised by the reviewers but we admit that we were unable to address all the concerns since some of them are obviously subjects for future debates. Only time will tell whether our dnaE-based grouping scheme is correct or not. In addition, for better written English, the final manuscript has been further revised by Edanz group editors*.

## Supplementary Material

Additional file 1 **Linear correlation between optimum growth temperatures and GC content among dnaE-based groups**. Click here for file

Additional file 2 **Common mutator genes and their resultant mutation patterns when mutated or defective**. Click here for file
