# Supplementary material for: On the molecular mechanism of GC content variation among eubacterial genomes
Source: Biol Direct. 2012 Jan 10;7:2. doi: 10.1186/1745-6150-7-2 (PMC3274465; doi:10.1186/1745-6150-7-2)
Supplement: Additional file 1 — Linear correlation between optimum growth temperatures and GC content among dnaE-based groups. [file 1745-6150-7-2-S1.DOC]

**Additional file 1: Linear correlation between optimum growth temperatures and GC content in dnaE-based groups.**

**
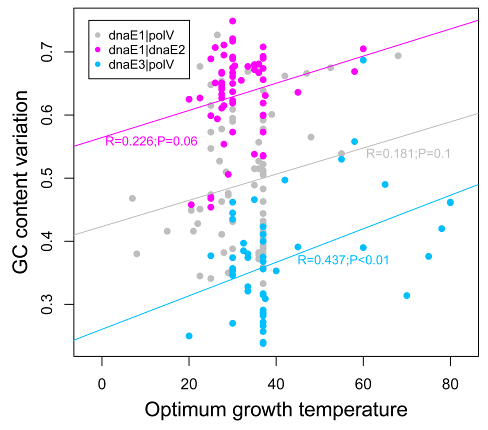
**
