# Supplementary material for: On the molecular mechanism of GC content variation among eubacterial genomes
Source: Biol Direct. 2012 Jan 10;7:2. doi: 10.1186/1745-6150-7-2 (PMC3274465; doi:10.1186/1745-6150-7-2)
Supplement: Additional file 2 — Common mutator genes and their resultant mutation patterns when mutated or defective. [file 1745-6150-7-2-S2.DOC]

**Additional file 2:** Common mutator genes and their resultant mutation patterns when mutated or defective.

| **Mutator Genes** | **Mutated/Defective** | **Magnitude** |
| --- | --- | --- |
| polC | - | - |
| dnaE1 | - | - |
| dnaE2 | - | - |
| dnaE3 | - | - |
| dnaQ/mutD | - | - |
| polA | - | - |
| mutT | AT-CG | 10^3^ fold |
| mutSHL,uvrD | - | - |
| mutY/M | GC-TA | 10^3^ fold |
| miaA | GC-TA | - |
| sodA/B | - | 40 fold |
| nth,nei | GC-AT | 2-8 fold |
| xthA,nfo | AT-TA | 4-9 fold |
| ung | GC-AT | 10-15 fold |
| vsr | GC-AT | - |
| ada,ogt | GC-AT | - |
| recA | GC-TA, AT-TA | 10-20 fold |

Note: “-” indicates outcome and magnitude unknown.
